# Vitamin D in Behcet’s Disease, a Brief Review of the Literature

**DOI:** 10.5152/eurasianjmed.2022.22300

**Published:** 2022-12-01

**Authors:** Mehmet Melikoglu, Mestan Sahin, Meltem Alkan Melikoglu

**Affiliations:** 1 Ataturk University School of Medicine, Dermatology, Erzurum, Turkey; 2 Department of Rheumatology, Ataturk University School of Medicine, Internal Medicine, Erzurum, Turkey; 3 Department of Rheumatology, Ataturk University School of Medicine, Physical Medicine and Rehabilitation, Erzurum, Turkey

**Keywords:** Behcet’s disease, Behcet’s syndrome, vitamin D, hydroxy vitamin D

## Abstract

Behcet’s disease is a chronic vasculitis of unknown etiopathogenesis. Serum vitamin D levels have been reported to be associated with a variety of inflammatory and autoimmune diseases one of which is Behcet’s disease. The previous studies about vitamin D in Behcet’s disease seem to be focused on 4 main categories; the studies evaluating serum vitamin D levels between patients with Behcet’s disease and controls, the studies evaluating serum vitamin D in the susceptibility and pathogenesis of Behcet’s disease, the studies evaluating serum vitamin D in clinical involvements and activity of Behcet’s disease, and the studies evaluating the effect of serum vitamin D replacement in Behcet’s disease.

The aim of this manuscript was to evaluate the results of the studies on serum vitamin D in Behcet’s disease and review the literature.

## Introduction

Behcet’s disease (BD) is a chronic vasculitis of unknown etiopathogenesis.^1^ Since the disease was first described in 1937 by Hulusi Behcet as a characteristic triad including oral and genital aphthous ulcers and relapsing iritis, its clinical spectrum was highly enlarged.^2-4^ The disease appears to be frequent in the Mediterranean region, Middle East, and Far East, mostly in Turkey and Japan.^5,6^

The cause and pathogenesis of the disease have not been clarified yet. However, genetic factors seem to be one of the major possible factors that take part in pathogenesis.^1,7,8^ Despite some genetic research, the mode of inheritance is not clear. On the basis of genetic factors, the interactions between environmental or other several factors have been suggested as causative factors.^9-11^

The immunological processes and oxidative stress have been investigated as actors in the disease’s development and progression.^12-16^ Endothelial cell injury may play an important role in the pathogenesis and immunopathology of this systemic vasculitis.^17-20^ Some adhesion molecules were also investigated as related factors.^21^

Today BD, with its highly enlarged clinical spectrum, is a multisystemic vasculitis. Vascular involvements are one of the basic characteristics of the disease.^22,23^ However, it can affect almost all organs and systems.^3,24-26^ In addition to the classical triad, dermatological, musculoskeletal, pulmonary, cardiovascular, gastrointestinal, and nervous system involvements were reported.^27^

Behcet’s disease may progress with relapses and remissions.^28^ There is no routine test to evaluate the activity of BD.^29^ Although some laboratory markers have been suggested to be associated with BD clinical activity, the assessments of a patient with BD are mainly clinical evaluations.^30,31^

As a chronic multisystemic vasculitis, BD may affect the quality of life (QoL) and related factors.^32^ It was reported that BD disease activity can affect QoL, and a patient’s impression of disease activity and joint involvement can be related factors to depression in this disease.^33,34^ Furthermore, 10% of cases may suffer from musculoskeletal pain, and fibromyalgia can be seen in these cases.^35^

Serum vitamin D has been defined to have a role in different processes, such as cell differentiation, proliferation, apoptosis, and hormonal and immune regulation of several systems.^36,37^ Vitamin D deficiency is common all around the world.^38^ The vitamin has been reported to be associated with several diseases in the literature. Its possible relation with infections has been widely reported.^39-43^ Also, there are some studies about vitamin D status in endocrinological, metabolic, and neurological disorders.^44-50^ Similarly, there are some investigations on vitamin D in some malignancies.^51-56^ Low vitamin D levels have been reported to be related to a variety of inflammatory and autoimmune diseases.^57-62^ The accurate mechanism underlying the connection between vitamin D and autoimmune diseases remains unclear. It may be reasonable that vitamin D might be an important factor in the modulating of immune responses in several processes.

There are also several studies investigating serum vitamin D in BD. The aim of this manuscript was to evaluate the results of the studies on serum vitamin D in BD and review the literature.

The previous studies about vitamin D in Behcet’s disease may be classified into 4 main categories:

- The studies evaluating serum vitamin D levels between cases with BD and controls.
- The studies evaluating serum vitamin D in the susceptibility and pathogenesis of BD.
- The studies evaluating serum vitamin D in clinical involvements and activity of BD.
- The studies evaluating the effect of serum Vitamin D replacement in BD.

## The Studies Evaluating Serum Vitamin D Levels Between Cases with Behcet’s Disease and Controls

There have been several investigations comparing the serum vitamin D status between cases with BD and controls. In a previous investigation comparing the levels of this vitamin in 32 cases with BD to 31 controls, significantly lower vitamin D levels were determined in cases than in controls (*P* < .001).^63^ No correlation was found between 25-hydroxyvitamin D levels and demographic findings, disease duration, or acute phase responses. Smoking, alcohol intake, and use of colchicine were found as the main predictors of vitamin D levels. Similar to these results, in a case-control study by Khabbazi et al.^64^ vitamin D levels were determined to be significantly lower in 48 cases with BD than in 47 controls.

On the contrary in a previous case-control study, significantly higher vitamin D levels were determined in cases of BD than in matched controls.^65^ Also, vitamin D levels were found to be lower in cases with active BD than in cases with inactive disease in this investigation. Faezi et al^66^ also aimed to compare the serum level of vitamin D in cases with BD to controls. They determined vitamin D deficiency in about 57% and vitamin D insufficiency in about 17% of cases with BD. Vitamin D deficiency was significantly more common in 112 controls than in 112 cases with BD (*P* < .001). The investigators showed no significant relationship between vitamin D level and disease duration, disease activity, Pathergy test, or HLA-B51 in cases with BD. So they concluded that vitamin D deficiency may be common among cases with BD; however, vitamin D deficiency was significantly more common in controls than in cases with BD in this study.

In a meta-analysis evaluating the linkage between vitamin D levels and BD in case-control studies, the authors found no statistically significant difference in vitamin D levels between cases with BD and healthy individuals.^67^

*Considering together the previous studies evaluating serum vitamin D levels between cases with BD and controls, the results seem to be conflicting. In addition to the methodological heterogeneity of the studies, the high prevalence of vitamin D insufficiency in the general population used as controls may limit the ability to achieve a conclusion. Therefore, further studies should be performed to directly address the comparison of vitamin D levels between cases with BD and controls.*

## The Studies Evaluating Serum Vitamin D in the Susceptibility and Pathogenesis of Behcet’s Disease

There have been a relatively large number of investigations evaluating the role of vitamin D in BD susceptibility and pathogenesis. Dal et al^68^ evaluated the vitamin D receptor (VDR) gene polymorphisms in the pathogenesis of BD in our population. Since they found significant differences in some genotypes between cases with BD and controls, they concluded that these gene polymorphisms may have a possible effect on BD pathogenesis. In another study on Iranian Azary cases with BD, the investigators aimed to evaluate the association between VDR gene polymorphisms and the susceptibility to BD and its clinical manifestations. They demonstrated the VDR f allele and f/f genotype were associated with BD in the Iranian Azari population.^69^

In another VDR gene study in BD, the investigators aimed to evaluate the methylation status of the VDR gene expression of cases with BD. The study concluded that expression of this gene decreased in cases with BD; however, no evidence was found about the regulation by a unique DNA methylation mechanism.^70^ In another previous study, VDR gene polymorphisms were investigated in cases with BD and cases with BD neurological involvement in a Turkish population.^71^ TaqI, FokI, and ApaI polymorphisms in the VDR were studied in cases with BD and the allelic and genotype distributions these polymorphisms were not found to be different among groups in our population except the higher frequency of ApaI A allele in controls than that in cases (60% versus 38.5%). Similarly, Karray et al^72^ investigated the relation between VDR gene polymorphisms FokI and BsmI with susceptibility to rheumatoid arthritis and BD in a Tunisian population. Although no significant relation was observed between the Bsm1 polymorphism and these diseases, the FokI F allele and F/F genotype were found to be significantly associated with BD and with the existence of vascular involvements.

The genetic polymorphisms of vitamin D metabolism were studied in ocular BD in a Chinese Han population.^73^ In this study, DHVR7 gene polymorphism among the single nucleotide polymorphisms of vitamin D family genes was found to be related to the susceptibility to ocular BD.

In this category of studies, Hamzaoui et al^74^ aimed to evaluate the disease characteristics related to vitamin D levels in cases with BD and its interaction with inflammatory processes. In this investigation, BD activity was found to be associated with lower vitamin D levels in cases with this disease. Furthermore, vitamin D status was associated with a reduction in Treg cells and a skewing of the Th1/Th2 balance toward Th1. These data pointed out that vitamin D was an important factor of T cell regulation reflecting its immunomodulatory effect.^74^ Do et al^75^ also aimed to search the immunomodulatory impact of vitamin D in BD, and they investigated the linkage between the Toll-like receptor (TLR) expression and the serum vitamin D status in BD. They found that serum vitamin D tended to be lower in active disease in BD, and the monocytes of the cases with active BD presented higher expressions of TLR2 and TLR4 than those of controls. The study concluded that the inflammation that may be triggered through TLR2 and TLR4 has an important role in BD pathogenesis and vitamin D might be a therapeutic option by modulating this process.

In contrast to other studies, in a study focused on the vitamin D status levels and its association with BD risk in large patient populations, data showed that genetically increased vitamin D level was associated with a higher risk of BD.^76^

In a previous meta-analysis about the linkages between VDR polymorphisms and susceptibility to BD, it was demonstrated that the association between FokI and ApaI polymorphisms in this gene and the disease risk might suggest the possible role of VDR in the BD pathogenesis.^77^

*Considering the possible role of vitamin D in the susceptibility to BD and in its pathogenesis, the results seem to be inconsistent among diverse populations. Therefore, further studies are needed to assess this possible association.*

## The Studies Evaluating Serum Vitamin D in Clinical Involvements and the Activity of Behcet’s Disease

In several studies, serum vitamin D levels were investigated as a potential factor in clinical involvements and disease activity in BD. In a previous study, the purpose was to assess serum vitamin D levels and to evaluate its association with disease activity, endothelial function, and carotid intima media thickness (CIMT) in cases with BD.

Although vitamin D insufficiency was found significantly more frequent in cases with BD than in controls, there was no significant association between this vitamin status and CIMT or endothelial function.^78^ In another study, Omar et al^79^ investigated the relationship between vitamin D levels, oxidative stress parameters, BD disease activity, and severity.^79^ An association was determined between lower vitamin D status and oxidative stress state in cases with BD and they concluded that vitamin D replacement may affect disease severity positively in these patients. In another study, it was aimed to evaluate vitamin D levels in Algerian cases with BD and its possible association with disease activity.^80^ Vitamin D deficiency was found to be associated with active BD in this study. The authors concluded that since this vitamin down-modulates nitric oxide production in cases with BD, vitamin D replacement might have an effect in the modulating inflammation process in BD.^80^ Ganeb SS et al^81^ also investigated the impact of vitamin D on disease measures in cases with BD. The authors found that serum vitamin D levels were significantly lower in cases with BD compared to controls. Furthermore, significant relationships were determined between vitamin D status and age, disease activity, and levels of acute phase responses (erythrocyte sedimentation rate [ESR] and c- reactive protein [CRP]) in cases with BD. They also mentioned that low vitamin D may lead to a predisposition to active disease, especially in older cases. In another study, Kul et al^82^ investigated vitamin D levels and bone mineral density in BD. The authors concluded that BD may cause a decrease in bone mineral density even if the disease has a subclinical inflammatory course, so vitamin D optimization should be targeted to prevent the development of secondary osteoporosis in these cases. In another study, it was aimed to search for a possible association between vitamin D and BD clinical manifestations or disease activity.^83^ Although significantly lower levels of vitamin D were found in cases with BD, no correlation was observed between BD activity and vitamin D levels in this investigation.^83^ In another study evaluating the association between vitamin D status and BD activity, similarly, no correlation was found between vitamin D deficiency and BD activity despite lower vitamin D in the BD cases compared to controls.

In a systematic review of the possible linkage between vitamin D status and clinical disease activity of BD, the data of 939 participants were pooled for their meta-analysis.^84^ With the analysis of these large number of cases, it was found that the serum vitamin D levels of the active BD cases were significantly lower than those in controls. On the basis of these results, the authors concluded that there was an association between vitamin D deficiency and BD activity. Finally, in a recent systematic review, it was also concluded that the current evidence may support the consideration that an optimized serum status of vitamin D would be associated with a substantially lower risk of active BD.^85^

*In terms of this category of studies evaluating serum vitamin D in clinical involvements and activity of BD, although there are results showing correlations between vitamin D and BD clinical involvements or activity, there are also some results that failed to determine such a relationship. This inconsistency may be due to the methodological heterogeneity of the investigations.*

## The Studies Evaluating the Effect of Serum Vitamin D Replacement in Behcet’s Disease

There were also some studies focused on the effect of vitamin D replacement in cases with BD. Güngör et al^86^ investigated the role of vitamin D on endothelial and immunological markers in BD and they showed some positive effects of vitamin D replacement on vascular dysfunction in these cases.

In a previous study which investigated the level of serum vitamin D and its possible linkage with endothelial function and CIMT in cases with BD, it was shown that carotid intima thickness measurements improved after vitamin D replacement therapy.^78^ In another investigation, the effect of vitamin D was evaluated in the inflammation process of the BD-like mouse model.^87^ Vitamin D improved BD-like symptoms by down-regulating the expression of TLR and pro-inflammatory cytokines in in vivo mouse models.

*The studies evaluating the effect of vitamin D replacement in cases with BD seem to be limited. Although some data are available in favor of vitamin D replacement in BD, further investigations are needed to directly address its contribution to BD since its effects are obvious in vitamin D insufficiency in the general population.*

In conclusion, since vitamin D seems to have a significant impact on the immune system modulation, its deficiency has been linked to several diseases one of which is BD. Although the methodological heterogeneity of the studies, the diversity of the study populations, and the high frequency of vitamin D deficiency in the general population may limit the ability to achieve a single conclusion, several studies pointed out linkages between vitamin D and several BD processes.
